# Experimental Evidence for Therapeutic Potentials of Propolis

**DOI:** 10.3390/nu13082528

**Published:** 2021-07-24

**Authors:** Priyanshu Bhargava, Debajit Mahanta, Ashish Kaul, Yoshiyuki Ishida, Keiji Terao, Renu Wadhwa, Sunil C. Kaul

**Affiliations:** 1 AIST-INDIA DAILAB, National Institute of Advanced Industrial Science & Technology (AIST), Tsukuba 305-8565, Japan; bhargava.priyanshu89@gmail.com (P.B.); debajit_m@yahoo.com (D.M.); ashish-kaul@aist.go.jp (A.K.); renu-wadhwa@aist.go.jp (R.W.); 2 DBT-APSCS&T Centre of Excellence for Bioresources and Sustainable Development, Kimin 791121, India; 3 CycloChem Co., Ltd., 7-4-5 Minatojima-Minamimachi, Chuo-ku, Kobe 650-0047, Japan; yoshiyuki.ishida@cyclochem.com (Y.I.); keiji.terao@cyclochem.com (K.T.); 4 Kaul-Tech Co., Ltd., Nagakunidai 3-24, Tsuchiura 300-0810, Japan

**Keywords:** honeybee, propolis, caffeic acid phenethyl ester (CAPE), artepillin C (ARC), biomedical properties, natural drug, traditional healthcare

## Abstract

Propolis is produced by honeybees from materials collected from plants they visit. It is a resinous material having mixtures of wax and bee enzymes. Propolis is also known as bee glue and used by bees as a building material in their hives, for blocking holes and cracks, repairing the combs and strengthening their thin borders. It has been extensively used since ancient times for different purposes in traditional human healthcare practices. The quality and composition of propolis depend on its geographic location, climatic zone and local flora. The New Zealand and Brazilian green propolis are the two main kinds that have been extensively studied in recent years. Their bioactive components have been found to possess a variety of therapeutic potentials. It was found that Brazilian green propolis improves the cognitive functions of mild cognitive impairments in patients living at high altitude and protects them from neurodegenerative damage through its antioxidant properties. It possesses artepillin C (ARC) as the key component, also known to possess anticancer potential. The New Zealand propolis contains caffeic acid phenethyl ester (CAPE) as the main bioactive with multiple therapeutic potentials. Our lab performed in vitro and in vivo assays on the extracts prepared from New Zealand and Brazilian propolis and their active ingredients. We provided experimental evidence that these extracts possess anticancer, antistress and hypoxia-modulating activities. Furthermore, their conjugation with γCD proved to be more effective. In the present review, we portray the experimental evidence showing that propolis has the potential to be a candidate drug for different ailments and improve the quality of life.

## 1. Introduction

The term propolis is derived from the Greek words pro, meaning ‘in front of’ or ‘at the entrance to’, and polis, meaning ‘community’ or ‘city’, and it describes a substance used to defend the hive. Propolis, also known as ‘bee glue’, is the most important ‘chemical weapon’ of honeybees. It is a brownish resinous material collected by bee workers from the flora (leaf and bark of tree species including birch, poplar, pine, alder, willow and palm) they live on or visit. Bees carry and accumulate the resinous exudates from these plants in their hives where they are modified by their enzymes into propolis, which is used as a construction material for the hives. It also prevents the spread of microbial (bacterial and fungus) infections and attacks from predators. Propolis was identified and used by humans since ancient times (since 300 BC) as a traditional medicine [1,2]. Egyptians benefited from the antiputrefactive properties of propolis in order to preserve their corpses from decomposition [3,4]. Propolis was historically recognized for its medicinal properties by the Greek and Roman physicians and was first employed as a mouth disinfectant and an antiseptic and healing natural drug in the treatment of wounds [3]. It has been listed as an official drug in London pharmacopeias of the 17th century and has since then been used as a popular and regular remedy, either in a pure form or in combination with other natural products in cosmetics/health industries [5]. Most recently, several research labs have investigated the bioactive constituents of propolis, a wide range of their bioactivities (including antioxidant, antibacterial, antifungal, anti-inflammatory, hepatoprotective and anticancer) and their mechanisms of action as nutritional, cosmetic and pharmaceutical benefits [6,7,8].

## 2. Propolis Constituents

Propolis has been classified into different types based on their geographic origin and plant source. The main widely known types include Poplar (New Zealand, Europe, North America, some regions of Asia), Green (Alecrim), Brazilian (Brazil), Birch (Russia), Red (Cuba, Brazil, Mexico), Mediterranean (Sicily, Greece, Crete, Malta) and Pacific (Okinawa, Taiwan, Indonesia) propolis. New Zealand and Brazilian green propolis are the two most prominent types that have attracted both scientific and commercial interest. In the past, the quality of propolis was determined based on the local varieties of bee species, botanical sources and climate conditions. In recent years, analytic technologies, namely high-performance liquid chromatography (HPLC), thin-layer chromatography (TLC), gas chromatography (GC), nuclear magnetic resonance (NMR), mass spectrometry (MS) and gas chromatography–mass spectrometry (GC-MS), have been extensively used to define chemotypes of propolis. With such highly refined analytic technologies, propolis constituents such as sugars, hydrocarbons and minerals have also been identified and regularly used for their profiling and branding.

More than 180 compounds, mainly polyphenols, have been identified as constituents of varieties of propolis originating from different geographical regions. Polyphenol content is often used as a criterion to evaluate the quality of propolis. The major polyphenols are flavonoids, phenolic acids, esters, phenolic aldehydes and ketones. Volpi et al. [9] reported 12 different flavonoids, pinocembrin, acacetin, chrysin, rutin, catechin, naringenin, galangin, luteolin, kaempferol, apigenin, myricetin and quercetin; two phenolic acids, cinnamic acid and caffeic acid; and one stilbene derivative, resveratrol, in propolis extracts. Terpenes (secondary metabolites) in propolis account for its resinous odor and are considered as a criterion to distinguish between different types of propolis and sometimes even as a marker of premium versus ordinary propolis. Other compounds in propolis include volatile oils and aromatic acids (5–10%), waxes (30–40%), resins, balms and pollen grains that offer the prime source of essential elements such as magnesium, nickel, calcium, iron and zinc. It is also enriched in biometabolites (amino acids, sugars, nucleic acids and lipids), minerals (Mg, Ca, I, K, Na, Cu, Zn, Mn and Fe), hydrocarbons (alkanes, alkenes, alkadienes, monoesters, diesters, aromatic esters, etc.) and vitamins (B1, B2, B6, C and E) that are essential for various structures and functions in living systems. Enzymes such as succinic dehydrogenase, glucose-6-phosphate, adenosine triphosphate and acid phosphate have been detected in a variety of propolis types. The chemical compositions of varieties of propolis from different geographical and botanical niches show striking variability, posing additional challenges in the application of propolis in health/cosmetic/pharmaceutical industries [10,11,12,13,14].

The appearance and physical properties of propolis also vary according to its geographical location and vegetation. It is generally dark green or brownish in color, hard at low temperature and soft at high temperature and melts at 60 to 70 °C; some kinds have melting temperature close to 100 °C. For commercial use, propolis extracts are often prepared with suitable solvents, such as ethanol, methanol, chloroform, ether and acetone, of which ethanol extract has been popular due to its high level of bioactivities. Several kinds of propolis products including dentifrices, lozenges, mouth rinses, creams, gels, cough syrups, wine, cake, powder, soap, chewing gums, tablets, candies, shampoos, chocolate bars, skin lotions and toothpaste are commercially available [15,16]. Furthermore, polyphenol-rich varieties of propolis from different geographical regions are commonly used as antioxidant and antiseptic remedies in traditional home-medicine systems.

A variety of extraction methods including hexane; alcoholic; and nonalcoholic mixtures of solvents such as polyethylene glycol (PEG)-400, water and olive oil have been used to generate propolis extracts. Interestingly, there were no large differences observed in the total content of phenolic compounds observed in different extracts. PEG–water extracts showed significant amounts of naringenin; galangin; kaempferol; ferulic, caffeic and p-coumaric acids; quercetin; and artepillin C. Many of these were tested to possess considerably similar levels of antimicrobial activity against *S. aureus*, *B. cereus*, *P. aeruginosa* and *K. pneumoniae* compared to the standard ethanol extract [17]. Ethanolic and hexane extracts of Brazilian propolis were tested on methicillin-resistant *Staphylococcus aureus* (MRSA) for antibacterial activity [18]. Initial comparison found more artepillin C (active component of Brazilian propolis) content in ethanol extracts as compared to hexane extracts. In addition, artepillin C showed bacteriostatic activity with membrane blebbing, resulting in antibacterial activity against *S. aureus* and anaerobic bacterium *Porphyromonas gingivalis* [19]. Other bioactive components, i.e., 3-prenyl-cinnamic acid allyl ester, 2-dimethyl-8-prenylchromene, kaempferide, drupanin and p-coumaric acid also showed antibacterial activity against *S. saprophyticus*, *L. monocytogenes* and *E. faecalis* [20]. Pinocembrin and apigenin, flavonoids found in Chilean propolis, showed significant antibacterial activity against *S. mutants* as compared to a mixture of polyphenols and chlorhexidine (MIC = 1.6 µg/mL) with MIC 1.4 µg/mL and 1.3 µg/mL, respectively [21]. Apigenin showed antibacterial activity against several Gram-negative bacteria, namely *P. aeruginosa*, *K. pneumoniae, S. enterica* serotype Typhimurium, *P. mirabilis* and *E. aerogenes* [22]. Quorum sensing is a bacterial communication mechanism used to express various survival or virulence traits to enhance resistance to various external stimuli. Propolis is rich in cinnamic acid and its derivatives, as they exist in the green parts of plants and flowers. They inhibit cell growth by damaging bacterial cell membranes and inhibiting ATPase, cell division and biofilm formation with anti-quorum sensing activity [23].

## 3. Propolis Bioactivities

As discussed in the above section, different types of propolis differ in their chemical constituents and bioactivities, which accordingly determines their therapeutic applications, including antibacterial, antioxidant, anti-inflammatory, antifungal, antiviral, cardioprotective, hepatoprotective, neuroprotective and anticancer applications.

### 3.1. Infections

Microorganisms (bacteria, viruses, fungus, protozoans and other parasites), the root cause of infectious human diseases, possess high genome instability and high adaptability to environmental conditions. Based on these features, infectious agents are rather difficult to treat with single-module synthetic drugs. The natural compounds and extracts derived from propolis (economical and easily available) have been proved to be effective and useful in not only treating, but also preventing infections.

#### 3.1.1. Antifungal Activity

Fungal infections are often caused by microscopic fungi through air, soil, water and plants. In the last few years, fungal diseases caused over 1.6 million deaths, and over a billion people suffer from fungal diseases annually. Fungal diseases can also cause major losses in agriculture and food production. Ota et al. [24] conducted efficacy testing of propolis for antifungal activity using 80 strains of Candida yeasts including *Candida albicans*, *Candida tropicalis*, *Candida krusei* and *Candida guilliermondii*. The antifungal activity of propolis was shown in the following order of sensitivity: *C. albicans* > *C. tropicalis* > *C. krusei* > *C. guilliermondii*. Antifungal activity of propolis has been demonstrated by in vitro assay for human dentinal tubules that are commonly infected with *Candida albicans*. Infected dental samples treated with propolis showed a reduction in infection as compared to the control group [25]. In another study, 105 human teeth were infected with *Candida albicans* for 2 days followed by treatment with propolis for 5 days. The treatment inhibited ~99% of fungal growth and was as effective as 2% chlorhexidine and alcoholic extract of *Azadirachta indica* [26].

Caffeic acid (an active component of propolis) showed remarkable antimycotic activity towards *Helminthosporium carbonum*, a pathogenic fungus that causes corn leaf blight disease. This fungus is a common inhabitant in dead or decayed leaves. Anti-*H. carbonum* activity is important to induce resistance or production of inhibitory compounds in maize as a defense mechanism. Major constituents of propolis such as 3-acetylpinobanksin, pinobanksin-3-acetate, pinocembrin, p-coumaric acid and caffeic acid have been shown to possess antifungal activity [27,28,29]. Pinocembrin was reported to possess activity against *Penicillium italicum*, a widely reported postharvest fungal pathogen affecting citrus fruits. *P. italicum* causes major menace through their spores that are airborne and appears as a fine powder. Infected fruits are completely covered by white mycelium followed by green and bluish spores, responsible for the decaying of citrus fruits. Pinocembrin has been shown to stop mycelial growth by inhibiting respiration and resulting in an imbalance in energy homeostasis. Based on these findings, pinocembrin has been proposed as a promising bioactive compound of propolis for the treatment of *P. italicum* infections on postharvest citrus fruit [30]. The flavonoids present in propolis have also been shown to possess considerable fungicidal activity against a variety of fungi strains including *C. pelliculosa*, *C. albicans*, *C. famata* and *C. glabrata* [31,32].

#### 3.1.2. Antibacterial Activity

In recent years, bacterial infections of crops and food products have attracted the attention of several health organizations. These microorganisms not only target crops but also spread several foodborne diseases, including food poisoning, fever and gastroenteritis. Propolis and its active components have also been shown to possess antimicrobial activity and boost the activity of conventional synthetic antimicrobial compounds [33,34]. Based on these reports, the antibacterial activity of propolis is often evaluated at two levels: (i) direct action on the microorganisms and (ii) stimulation of the immune system. Several studies showed that propolis and some of its flavonoid components inhibited bacterial (*B. subtilis*) motility by dissipating the membrane potential and uncoupling the energy-transducing cytoplasmic membrane [35]. The toxic effect of propolis was therefore concluded as a direct effect on membrane permeability and membrane potential. Propolis was also found to be synergistically effective with most of the antibiotics except polymyxin B and nystatin. It was reported to affect a wide range of Gram-positive rods and have limited activity against Gram-negative bacilli. Bulgarian propolis was shown to be active against most anaerobic bacterial strains and other pathogens, including Clostridium, Bacteroides and Propionibacterium species [36]. Furthermore, xanthochymol and oblongifolin found in red propolis were reported to possess antimicrobial activity against multidrug-resistant bacteria [37]. The differential activity towards Gram-positive and Gram-negative bacteria was attributed to the structural differences in the outer bacterial membrane and the production of hydrolytic enzymes in Gram-negative bacteria that cause degradation of the active component of propolis [38]. *Helicobacter pylori*, a major factor for gastrointestinal illness, contains the enzyme peptide deformylase that catalyzes the removal of the formyl group from the N-terminus of nascent polypeptide chains. Investigations on the absorption spectra and crystal structure have shown that CAPE, an active component of propolis, is a competitive inhibitor of peptide deformylase. It can block the substrate–active site interactions. As the action of this enzyme is essential for *H. pylori* survival, propolis CAPE has been proposed as a promising therapeutic drug candidate [39]. Feeding of ethanolic extract of propolis (300 mg/kg body weight) for 30 days was shown to be most effective against Salmonella in infected BALB/c mice [40]. Furthermore, propolis enhanced the activity of antimicrobial drug cefixime (acts by penetrating the monocytes and causes inhibition of *Salmonella typhimurium* growth), showing significant inhibitory effect on the growth of Salmonella in just 5 days [40]. These data have suggested the synergistic activity of propolis and conventional synthetic antimicrobial agents.

#### 3.1.3. Antiviral Activity

Viruses, the small infectious agents that replicate only inside the living host cells, cause several kinds of short-term (e.g., common cold, influenza, chickenpox, smallpox, measles and cold sores) and long-term (e.g., chronic hepatitis, rabies, Ebola virus disease, AIDS and several types of cancers) ailments. Several flavonoids have been shown to possess inhibitory activity towards viruses such as herpes simplex virus (HSV-1 and HSV-2), Sindbis virus, parainfluenza-3 virus, human cytomegalovirus and dengue virus type 2 [41,42,43]. It has been observed that flavonoids are more active than flavones, and the synergism of both compounds possibly presents better biological activity against various ailments as compared to individual substances [44]. Propolis contains a large amount of 3-methyl-but-2-enyl caffeate, isopentyl ferulate and moronic acid constituents. Influenza A (H3N2) virus, a respiratory pathogen, evades human immunity, preliminarily by acquiring antigenic changes in the hemagglutinin (HA). Isopentyl ferulate, a constituent of propolis, was shown to inhibit the infectious titer and activity of influenza virus A1 Hong Kong (H3N2) in vitro [45]. Herpes simplex virus (HSV-1) and HSV-2 both have been reported to cause oral and genital lesions due to autoinoculation. A minor constituent of propolis, 3-methyl-but-2-enyl caffeate, isolated from poplar buds was shown to possess anti-herpes simplex virus type 1 activity in in vitro assays. Addition of propolis at 6.25 μg/mL to a culture medium of infected Vero cells caused an effective reduction in the virus titer [46]. In a randomized study on a group of 90 men and women with recurrent chronic genital herpes simplex virus (HSV) type 2, ointment of Canadian propolis was identified as more protective than acyclovir and placebo ointments [5]. The replication of HSV-1 and HSV-2 was significantly suppressed in the presence of 25, 50 and 100 μg/mL of Hatay propolis. The synergistic effect of Hatay propolis with acyclovir was found prominent on both HSV-1 and HSV-2 viruses [47].

In developing countries, acquired immunodeficiency syndrome (AIDS) is a pronounced cause of death. The virus destroys the T cells after invasion and replicates inside the host cells. Most conventional drugs are nucleoside-based and carry limitations including adverse side effects, resistance and toxicity. Several flavonoids containing natural products and caffeic acid derivatives such as chicoric, rosmarinic and lithospermic acids have been shown to be active against HIV [48]. Anti-HIV activity in H9 lymphocytes treated with triterpenoids, melliferone, moronic acid, anwuweizonic acid, betulonic acid and four known aromatic compounds isolated from Brazilian propolis were evaluated. Moronic acid showed significant anti-HIV activity (EC50 < 0.1 μg/mL) [49]. Propolis has been shown to inhibit HIV expression in CD4+ lymphocytes and microglial cell cultures in a concentration-dependent manner.

Canine distemper virus (CDV) is closely related to the measles virus, bovine pestivirus, small ruminant pestivirus and phocine distemper virus. It causes multisystem disease in dogs and other carnivores [50]. Mexican propolis and its three active pure flavonoids quercetin, naringenin, and pinocembrin were tested for antiviral (CDV) activity in CCL-81 cells. The expression of virus nucleoprotein gene and cell viability measured to evaluate antiviral activity endorsed that propolis and quercetin caused a significant decrease in gene expression and increase in host cell viability, whereas naringenin and pinocembrin failed to stop viral infection [51].

The rhinovirus (HRV) is the most common virus and the predominant cause of the common cold. Kaempferol and coumaric acid, active ingredients of Brazilian propolis, were found to be effective for treating HRV-2, HRV-3 and HRV-4 viruses in a HeLa cell culture model. It was hypothesized that kaempferol and coumaric acid may inhibit viral entry into the cells, generating protection from viral destruction and abating virus replication [52].

The recent global pandemic of coronavirus disease 2019 (COVID-19), caused by a novel coronavirus (severe acute respiratory syndrome coronavirus 2 (SARS-CoV-2/2019-nCoV)) lacks treatment modalities and has triggered repurposing of the existing natural drugs, including propolis, that have been trusted for antimicrobial and antiviral activities in traditional systems of home medicine. Several computational and molecular docking studies have shown that the flavonoids in propolis (e.g., rutin, naringin, caffeic acid phenyl ester, luteolin and artepillin C) have the potential to inhibit viral spike fusion to the host cells. Interestingly, ethanolic extract of propolis and propolis liposomes have been shown to inhibit (i) structural proteins of SARS-CoV-2 in vitro and (ii) SARS-CoV-2 infection in Vero E6 cells when used in combination with naringin. Of note, nanocarrier-mediated delivery of propolis extracts enhanced their antiviral activity. In a preclinical study, COVID-19 patients receiving green Brazilian propolis showed earlier recovery of symptoms and viral clearance as compared to the group receiving standard treatment [53]. Several bioactive compounds in propolis have been predicted to possess inhibitory effects on the entry of virus to host cells by blocking the host cell receptors (ACE2, TMPRSS2), PAK1 signaling pathways and immunoregulation of proinflammatory cytokines (including a reduction in IL-6, IL-1 beta and TNF-alpha) [54]. Furthermore, propolis has been shown to be effective in the treatment of various comorbidities (respiratory diseases, hypertension, diabetes and cancer) that are high mortality risk factors for COVID-19 patients [55]. In an in silico study, binding constants of 10 flavonoids commonly found in propolis (caffeic acid, caffeic acid phenethyl ester, chrysin, galangin, myricetin, rutin, hesperetin, pinocembrin, luteolin and quercetin) to ACE2 receptor were investigated by molecular docking. Rutin showed the highest high binding energy (–8.04 kcal/mol), followed by myricetin, quercetin, caffeic acid phenethyl ester and hesperetin, suggesting their inhibitory potential for COVID-19 [56]. Khayrani et al. [57] identified five compounds (namely glyasperin A, broussoflavonol F, sulabiroins A, (2S)-5,7-dihydroxy-4′-methoxy-8-prenylflavanone and isorhamnetin) from Sulawesi propolis that have the potential to inhibit the binding of ACE-2 and SARS-CoV-2. Interestingly, the docking score of each of the test compounds was favorable as compared to a known potent inhibitor (MLN-4760).

On the other hand, in silico studies investigated the binding potential of bioactive compounds of propolis to SARS-CoV-2 proteins (Mpro and S2) that are essential for its replication. Shaldam et al. [58] reported the binding affinity of 14 selected phenolics and terpenes found in honey and propolis against the Mpro and RNA-dependent RNA polymerase (RdRp) enzymes of the SARS-CoV-2 virus. Of these compounds, p-coumaric acid, ellagic acid, kaempferol and quercetin showed the strongest interactions. In another study, four compounds (neoblavaisoflavone, methylophiopogonone A, 3′-methoxydaidzin and genistin) showed binding affinity similar to that of the positive control nelfinavir, thus placing them as antiviral drug candidates [59]. Two compounds from Sulawesi propolis, glyasperin A and broussoflavonol F, were shown to be potential drug candidates for COVID-19 based on their high binding affinity to the catalytic sites of Mpro [60]. Kumar et al. [61] also reported that the binding affinity and energy of caffeic acid phenethyl ester (CAPE) to the substrate-binding domain of Mpro were comparable to those of a positive control, N3 protease inhibitor. Refaat et al. [62] reported in silico analysis where rutin and CAPE were shown to possess the highest affinity to viral targets, 3CL-protease and S1 spike protein. They also developed a liposomal formula of propolis that exhibited significantly enhanced inhibition of viral replication as comparable to the viral inhibitory effect of the potent antiviral drug remdesivir. These recent reports have rejuvenated the interest in the bioactivities of propolis and its use as a valuable nutraceutical and pharmaceutical ingredient.

#### 3.1.4. Inflammation

Inflammation is largely defined as a biological response to an altered environment categorized as stress (exogenous or endogenous) that causes cell/tissue damage, triggered by physical (burns, bruises, radial damage, etc.), chemical (acid, alkali, allergens, etc.) and biochemical (parasites, microorganisms, endotoxins) factors. It is often divided into acute and chronic. In acute inflammation, immune system cells are activated and migrate to the site of damage and release growth factors, cytokines, reactive oxygen species (ROS) and reactive nitrogen species (RNS). Chronic inflammation occurs whenever the acute inflammation is not successfully resolved. The inflammatory condition has been shown to play a critical role in the pathogenesis of diseases, namely asthma, atherosclerosis, cancer, Alzheimer’s and Parkinsonism [63,64]. A broad range of signaling pathways has been reported to be associated with inflammation, namely nuclear factor kappa B (NFk-B), activator protein (AP)-1, peroxisome proliferator-activated receptor (PPAR) and nuclear factor erythroid 2-related factor 2 (Nrf2) transcription factors; mitogen-activated protein kinases (MAPKs); protein tyrosine kinases (PTKs); tyrosine phosphatidylinositol 3-kinase (PI3K)/Akt; and the ubiquitin–proteasome system. The identification and characterization of phenolic compounds that modulate signaling events involved in inflammation are important not only to validate the use of dietary phenolics in human health but also to find new potential molecules that serve as templates for the further structural development of safe and effective anti-inflammatory drugs. The anti-inflammatory properties attributed to the phenolic compounds are often related to their antioxidant activity in addition to the modulation of specific inflammation-signaling pathways [65].

Several studies have shown that flavonoids, polyphenolic compounds present in propolis, possess anti-inflammatory capability [65,66]. The main reported mechanisms of anti-inflammatory activity of propolis include (i) inhibition of cyclooxygenase (COX) and subsequent inhibition of prostaglandin biosynthesis, (ii) free radical scavenging activity, (iii) inhibition of nitric oxide synthesis, (iv) reduction in inflammatory cytokine levels and (v) immunosuppressive activity [67,68,69]. Immunomodulatory assays including tests with positive controls, such as lipopolysaccharide (LPS), concanavalin A (CON A), phorbol myristate acetate (PMA) or cytokines (IFN-γ), have been performed to assess the efficacy of propolis as an immunomodulator. The administration of 200 mg/kg ethanolic extract of Brazilian propolis to mice for 3 days enhanced the innate immunity, activated the immune response by upregulating the expression of toll-like receptors TRL-2 and TRL-4 and the production of proinflammatory cytokines interleukin IL-2 and IL-6 by macrophages and spleen cells, contributing to the recognition of microorganisms and activation of lymphocytes by antigen-presenting cells [70]. The effects of propolis extracts were investigated in rat models of inflammation [71]. The arthritis index in the chronic inflammatory animal model was suppressed upon treatment with propolis extract (50–100 mg/kg/day). In addition, physical weakness due to chronic disease state was improved in the propolis-treated group in a dose-dependent manner. It was suggested that the anti-inflammatory activity of propolis in both chronic and acute inflammation might be mediated by its inhibitory effect on prostaglandin production [71]. Several studies have demonstrated that propolis acts as a potent anti-inflammatory agent for chronic and acute inflammation conditions [72]. Propolis has also been shown to act on the nonspecific immune response by releasing hydrogen peroxide and inhibiting the production of nitric oxide in a dose-dependent manner [73]. Several studies have suggested that propolis extracts possess antioxidant activity due to their high content of polyphenols, flavonoids, caffeic acid, ferulic acid, CAPE and kaempferol. They block lipid peroxidation and protect cells from the damage caused by free radicals and overabundance of inflammation [73,74].

In a human study, early on, the administration of propolis capsules (500 mg) for 2 weeks showed spontaneous and LPS-induced increase in proinflammatory cytokine secretion capacity of peripheral blood leukocytes [75]. Another human study has reported the prolonged effect of propolis supplementation on redox state. Male population supplemented with propolis demonstrated a reduction in free-radical-induced lipid peroxidation as well as an increase in activity of superoxide dismutase [76]. Most recently, Chinese and Brazilian propolis types were shown to alter lipoglycan, endotoxin-based inflammatory cascade and nuclear factor kappa B (NF-kB) function [77,78,79]. In most studies, the most popular bioactive compound of New Zealand propolis, CAPE, was shown to possess remarkable anti-inflammatory properties. It showed immunosuppressive activity by inhibiting the early and late events of T-cell activation and the consequent release of cytokines. Treatment with propolis led to a reduction in pulmonary inflammation and mucus production. It caused an increase in in vitro differentiation and in vivo frequency of lung myeloid-derived suppressor cells (MDSC) and CD4^+^/Foxp3^+^ regulatory T cells, suggesting its potential in the treatment of allergies and asthma [68]. It was also reported to inhibit myeloperoxidase activity, NADPH oxidase, ornithine decarboxylase, tyrosine-protein kinase and hyaluronidase in guinea pig mast cells [1]. Several bioactive compounds other than CAPE have been assigned anti-inflammatory activity. These include pinocembrin, chrysin and artepillin C [80,81,82,83].

### 3.2. Liver Function

The liver is a large glandular organ comprising 1/50 of the total weight of the adult body. It synthesizes and secretes bile, lipoproteins and plasma proteins, including clotting factors. It maintains a stable blood glucose level by taking up and storing glucose as glycogen (glycogenesis), breaking this down to glucose when needed (glycogenolysis) and forming glucose from non-carbohydrate sources such as amino acids (gluconeogenesis). In conjunction with the spleen, the liver regulates the destruction of dying red blood cells and the reclamation of their constituents. Hepatotoxicity of drugs or drug-induced liver injury (DILI) is a major health problem accounting for more than 50% of acute liver failure, including that caused by overdosing of drugs. The mechanism of DILI involves two pathways, namely direct hepatotoxicity and adverse immune reactions. DILI is initiated by the bioactivation of drugs to chemically reactive metabolites having the ability to interact with cellular macromolecules leading to a variety of anomalies including protein dysfunction, lipid peroxidation, DNA damage and oxidative stress. Such impairments in subcellular structures and functions, accumulation of macromolecular damage and loss of energy homeostasis often culminate in cell death and possible liver failure. Early diagnosis and withdrawal of the suspected medication is the mainstay of treatment of DILI. Therapies developed along the principles of synthetic medicine are often limited in efficacy, carry the risk of adverse effects and are often expensive, especially for the developing world. Therefore, treating liver diseases with plant-derived compounds has attracted research and clinical interest [84,85,86].

Several studies have addressed the mechanism of the hepatoprotective activity of propolis and its active ingredients. Propolis extracts exhibit significant cellular recovery, as evidenced by the reversal of various biochemical indices and histopathology, altered due to CCl4 toxicity. Owing to the antioxidative potential, propolis has been assigned clinical importance and is useful for the treatment of metabolic syndrome and its associated chronic diseases [66]. Early on, using D-GalN-induced hepatic injury model, it was shown that ethanol extract of Brazilian and Cuban propolis types possess strong hepatoprotective activity against paracetamol- and CCl4-induced liver injury in mice and rat models [87]. Ethanolic extracts of propolis also showed protective effect against alcohol-mediated liver injury by preventing the elevations of total cytochrome P-450 enzymes, NADPH-dependent cytochrome C reductase, aniline hydroxylation, 7-ethoxyresorufin (7-ERH) hydroxylation, 7-penthoxyresorufin (7-PRH) hydroxylation and lipid peroxidation. A study on Brazilian propolis reported its protective effect against hepatic oxidative damage in rats with water-immersion restraint stress (WIRS) in comparison with vitamin E (VE) [88]. Exposure of vehicle-treated rats to 6 h of WIRS caused liver damage as indicated by levels of serum alanine aminotransferase and aspartate aminotransferase; increased hepatic liquid peroxide and NO(x) content and myeloperoxidase activity; and decreased hepatic non-protein SH, ascorbic acid content and superoxide dismutase activity. Rats on fast were orally administered with ethanol extract of Brazilian green propolis (BPEE10, 50 or 100 mg/kg) and VE (250 mg/kg) at 30 min before the onset of WIRS. Prior administration of BPEE (50 or 100 mg/kg) or VE to the stressed rats protected against the hepatic damage and attenuated the increased hepatic lipid peroxide, NOx and myeloperoxidase activities. These results indicate that BPEE protected against hepatic oxidative damage in rats exposed to WIRS, possibly through its antioxidant and anti-inflammatory properties.

Some studies have also reported the protective effect of CAPE in various hepatotoxicity models. Intraperitoneal (i.p.) administration of CAPE to 12-week-old female Wistar albino rats (10 μmol/kg body mass; 6 days), prevented cisplatin-induced oxidative damage in their liver by reducing ROS production and increasing antioxidative enzyme (such as SOD, GSH-Px and CAT) activities, signifying the hepatoprotective effect of CAPE [89]. CAPE (10 μmol/kg, i.p.) treatment for 2 weeks also showed protective effects against cholestatic oxidative stress and liver damage in common bile duct ligated rats [90]. It was found that the treated cells, in bile duct ligated rats, exhibit a reduction in the level of malondialdehyde (MDA) and an increase in superoxide dismutase (SOD) and glutathione peroxidase (GPx) enzymes in liver tissues. Based on these data, the hepatoprotective effect of CAPE was attributed to its antioxidative potential. Treatment with CAPE (10 mg/kg body mass; 10-days, i.p.) to Wistar albino rats revealed protective effects on the aflatoxin B1-induced hepatoxicity by modulating free radical production. In addition, it also caused a reduction in total oxidant capacity (TOC) and glutathione S-transferase activities [91]. CAPE-treated aged rats also showed better ultrastructure of liver tissues and an increase in tissue catalase (CAT) activity, suggesting its potential in delaying age-related hepatocellular alterations [92].

### 3.3. Brain Function

The brain is the most complex organ of the human body, both in structural and functional aspects. The neuron, its functional unit, is a specialized cell designed to transmit information between different parts of the brain and to other organs. It controls the functioning of all organs and all aspects of our cognitive behavior (thoughts, memory, speech, physical reaction and response). Aging, a natural and indispensable life phenomenon associated with functional decline and adaptability represents cumulative deteriorative changes in macromolecules that are the by-products of normal cell and tissue functions. These are highly impacted by internal and external stress factors. Most stresses evoke adaptive responses. It has been established that the adaptability to stress declines with age. Furthermore, exposure to severe or chronic stressors dysregulates adaptive and repair pathways and results in an accelerated aging phenotype. Degenerative changes in neurons in response to aging and metabolic, ionic and oxidative stresses result in protein aggregation, perturbation of energy homeostasis and mutations in nucleic acids [63]. Stress has been shown to cause apoptotic death of neurons and contributes significantly to neurodegenerative disorders. Often, these changes are connected to old-age-associated pathologies including loss of memory, amnesia, Alzheimer’s disease (AD) and Parkinson’s disease (PD) [63].

A large variety of studies using different experimental models have shown that natural products play a vital role in the protection of neuronal cells from neuroinflammation and oxidative stress, associated with normal and chronic age-related diseases. A diverse array of bioactive nutrients, present in natural products, have been predicted to play a significant role in the prevention and cure of various neurodegenerative disorders. Interestingly, polyphenols and flavonoids present in plants and other natural sources are becoming popular as health supplements. These compounds can interact with reactive oxygen and nitrogen species and terminate deteriorative chain reactions involved in oxidative and inflammation signaling involved in chronic diseases. For example, epigallocatechin-3-gallate (EGCG) polyphenol has been shown to provide a neuroprotective effect and the ability to resist amyloidosis. It showed a protective effect on damaged nerves, inhibited neuronal apoptosis and inhibited oxidative stress injury after middle cerebral artery occlusion by modulating PI3K/AKT/eNOS pathway [93]. Several preclinical and clinical studies have also revealed the importance of natural compounds such as resveratrol (polyphenol) and curcumin in stress protection against oxidative stress-induced neuronal damage, lipid peroxidation and behavior changes [94,95].

Propolis has also been reported to possess neuroprotective properties in vitro and in vivo through its antioxidant, anti-inflammatory and immunomodulatory effects [65,66,73,74,96]. Brazilian green propolis has been shown to protect SH-SY5Y neuroblastoma cells from neurodegenerative damage by upregulating brain-derived neurotrophic factor (BDNF) and activity-regulated cytoskeleton-associated protein (ARC), proteins involved in memory [97]. In this study, the pretreatment of SH-SY5Y cells with propolis extract significantly attenuated hydrogen peroxide induced toxicity and reduced ROS and 8-oxo-2′-deoxyguanosine levels (markers of oxidative damage in mitochondria and DNA, respectively). Brazilian yellow propolis, rich in triterpenoids, has been shown to elicit anxiolytic and antidepressant effects at low doses (~1 mg/kg). Ethanol extract of yellow propolis at 30 mg/kg was shown to improve cognitive function in rats without any sedative effects [98]. Artepillin C, a major component of Brazilian propolis, was also found to induce neurite outgrowth in rat PC12m3 cells in which nerve growth factor (NGF)-induced outgrowth is impaired. By molecular analyses, it was concluded that artepillin C induced activation of p38MAPK through the ERK signaling pathway, responsible for the neurite outgrowth [99]. These findings suggested the neuroprotective role of propolis and its therapeutic potential for the prevention of age-related cognitive impairment and brain pathologies. Propolis phenols are reported to possess selective permeability across the blood–brain barrier (BBB), and their systematic elimination limits the therapeutic efficacy with regard to optimal brain function [100]. On the other hand, several studies have suggested that CAPE and some other propolis derivatives are capable of crossing the BBB in rat models [101]. CAPE was shown to provide neuroprotection by inhibiting NF-kB function and reducing the release of proinflammatory miRNAs (miRNA-125b, miRNA-146a and miRNA-155) from primary human neuronal glial cells stressed with Alzheimer’s disease (AD) derived extracellular fluid [102]. This finding suggested that CAPE acts as an anti-NF-kB agent and could be a potential natural therapeutic drug candidate for the treatment of AD. In another study, CAPE was examined for its effects on oxidative tissue damage in experimental autoimmune encephalomyelitis (EAE), (a condition when the body’s immune system mistakenly attacks healthy brain cells) in rats [103]. Treatment with CAPE significantly inhibited reactive oxygen species (ROS) production induced by EAE and later ameliorated clinical symptoms in rats, possibly through its anti-inflammatory effect by suppression of NF-kB at the transcriptional level and catalytic activity of inducible nitric oxide synthase. One of the propolis flavonoids, pinocembrin, has also been shown to protect SH-SY5Y neuronal cells from glutamate stress by inhibition of cytochrome C and apoptotic proteins such as BAX and p53 [104]. Pinocembrin was also found to cross the BBB and alleviate BBB injury in a global cerebral ischemia/reperfusion model in rats [105]. These studies have supported the use of propolis for brain health, especially to protect against age- and stress-related anomalies.

### 3.4. Cardiac Function

The heart, an essential organ for pumping blood to the rest of the body, is controlled by a high degree of coordination of electrical signals that start in the right atrium. Aging is associated with a critical reduction in the control of such electrical signals and is often associated with progressive decline in numerous physiological processes, increased risk of heart attack and other organ pathologies. It has been well established that the free radicals produced during normal metabolic processes damage subcellular structures and macromolecules, including DNA, proteins, lipids and carbohydrates, and contribute to age-related tissue dysfunctions. Aging and oxidative stress have remarkable effects on the heart, leading to an increase in various cardiovascular diseases (CVDs), namely cardiomyopathy, hypertrophy, atherosclerosis, hypertension, myocardial infarction and stroke. Free radical species such as singlet oxygen, hydrogen peroxide and hydroxyl radical produced by the partial reduction of oxygen are highly unstable, reactive and toxic for all tissues and can severely perturb heart functioning. Studies on isolated perfused hearts revealed that even brief exposures to oxygen radicals lead to a decrease in high-energy phosphates and a loss of contractile function and cause structural abnormalities [106]. Several studies have documented oxidative stress as a root cause of insulin resistance, β-cell dysfunction, impaired glucose tolerance and cardiotoxicity.

Natural products offer unique structural and chemical diversity and have been shown to alleviate oxidative stress-induced diseases, including cardiovascular diseases (CVDs), by regulating the NRF2/antioxidant-responsive element (ARE) pathway [107]. Typically, natural products consist of several constituents that perform their actions independently or through synergistic complementarity, making them multitarget drugs. Epidemiological studies have demonstrated that nutritional habits such as the consumption of food rich in natural bioactive substances have been associated with a longer life expectancy and a significant decrease in the incidence and prevalence of CVD [108]. In a recent report, phenolic compounds derived from natural products olive oil (hydroxytyrosol) and red wine (resveratrol) have been reported to enhance nuclear translocation of Nrf2 (a protein involved in oxidative stress management) and reduce the expression of miRNA-146a (a noncoding regulator of inflammation response). Kubota et al. [109] examined the changes in cardiovascular parameters of hypertensive rats following a 4-week diet of Brazilian propolis extracts and found a significant reduction in systolic blood pressure, suggesting their antihypertensive effect. Molecular characterization revealed that tri-caffeoylquinic acids (CQAs: 3,4-diCQA, 3,5-diCQA and 3,4,5-triCQA), characteristic components of ethanol extract, at a dose of 10 mg/kg showed good antihypertensive activity and hence were recommended for the prevention and treatment of hypertension. In a study on mice on a cholesterol-rich diet, it was revealed that all types of propolis (red, green or brown) can successfully diminish cholesterol and elevated high-density-lipoprotein cholesterol concentrations and sclerotic lesions [110]. When low-density lipoprotein (LDL) receptor knockout mice (LDLr-/-) were treated with Brazilian red propolis (250 mg/kg/day), they showed decreases in the levels of triacylglycerol (TAG), total cholesterol and non-high-density-lipoprotein cholesterol [110]. An ethanolic extract of Brazilian red propolis has been reported to enhance ATP-binding cassette transporter gene-1 (ABCA1), a regulator of cellular cholesterol promoter activity in THP1 macrophages [111]. In addition, in vivo data in C57BL/6 mice treated with 50 mg/kg extract once a day for 4 weeks supported the in vitro findings and showed an increase in plasma HDL, but not LDL, cholesterol. Taken together, in vitro and in vivo studies have suggested propolis as a potential candidate for the prevention of atherosclerosis [112]. Administration of propolis in diabetic rats led to a decrease in the levels of blood glucose (FBG), fructosamine (FRU), malonaldehyde (MDA), nitric oxide (NO), nitric oxide synthetase (NOS), total cholesterol (TC), triglyceride (TG), low-density-lipoprotein cholesterol (LDL-C) and very low-density lipoprotein cholesterol (VLDL-C) in serum of fasting rats. The data suggested that propolis could control and modulate blood glucose, metabolism and blood lipids, leading to a decrease in output of lipid peroxidation, and scavenge the free radicals in rats with diabetes mellitus [113]. Doxorubicin-induced myocardiopathy is a consequence of free-radical-induced oxidative stress. The effect of intraperitoneal administration of propolis (50 and 100 mg/kg) was studied on cardiomyopathy induced by doxorubicin (10 mg/kg, i.v.) in rats. Thiobarbituric acid reactive substances (TBARS), serum creatine phosphokinase (CK), aspartate aminotransferase (AST) and blood and tissue glutathione (GSH) in the heart are estimated to assess the status of heart muscle. It was observed that the doxorubicin treatment elevated the levels of CK, AST, GSH and TBARS as a result of cardiotoxicity; however, pretreatment with propolis significantly reduced the levels of these parameters [114].

Flavonoids present in propolis are iron chelators and can therefore decrease the iron-dependent production of free radicals. Thus, chelation raises the level of scavenging activity of flavonoids. Pinocembrin, a flavonoid from propolis, is shown to affect cardiovascular diseases based on its ability to regulate apolipoprotein E (ApoE; a protein involved in the metabolism of fats) and reduce rho kinase (regulates shape and movement of cells) [115]. It was demonstrated that the combination treatment of simvastatin (a lipid-lowering medication) with pinocembrin for 14 weeks significantly reduced serum lipid levels, improved endothelial function and reduced atherosclerosis symptoms in ApoE-/- mice [116]. Platelet aggregation has been shown to contribute to atherosclerosis and atherothrombosis, complex multifactorial chronic inflammatory diseases initiated by the accumulation of fat and other macromolecules in the arterial walls. CAPE (15 and 25 µM) significantly inhibited collagen-stimulated platelet aggregation [117]. In addition, CAPE was also shown to protect the cardiac tissues against oxidative stress induced by hyperthyroidism in rats [118]. It was reported that CAPE exhibited cardioprotective effects in short-term myocardial ischemia in rats through the reduction in xanthine oxidase (XO) and adenosine deaminase (ADA) activities and antioxidant effects.

### 3.5. Antioxidant Activity

Living cells require oxygen to produce energy. It is a substrate for oxidative phosphorylation and ATP generation via the electron transport chain in mitochondria. Reactive oxygen species (ROS) are the indispensable product of this life-sustaining process. These are highly reactive molecular entities that cause structural and functional deteriorative changes and are regulated by the enzymatic and nonenzymatic antioxidant cellular defense mechanisms. The primary antioxidant enzymes are superoxide dismutase (SOD), catalase (CAT) and glutathione peroxidase (GPx). Age-associated decline in antioxidative enzymes has been shown to yield accumulation of oxidative damage as measured by the increase in the level of reactive oxygen species (ROS) and reactive nitrogen species (RNS). Mitochondria are the main organelles responsible for the production of ROS during physiological and pathological states [119]. Polyphenols, lignans, stilbenes, flavonoids, phenolic acids and derivatives of cinnamic (e.g., ferulic, coumaric and caffeic) and benzoic acids (e.g., gallic acids and hydroxybenzoic acids) have been shown to possess physiologically relevant antioxidant properties. The antioxidant capacity of propolis has also been related to some of its physiological effects, including chemoprevention. Cuban red propolis showed a protective effect against allyl alcohol induced liver injury in a mouse model [87]. The flavonoids in propolis are powerful antioxidants, capable of scavenging free radicals, and protect cells from a variety of macromolecular damage. In 2005, de Lima et al. showed that the aqueous extract (administered orally at 0.01%, 0.03%, 0.1% and 0.3% in the drinking water; approximately equivalent to 12, 34, 108 and 336 mg/kg body weight/day dose) of Brazilian propolis was able to protect against 1,2-dimethylhydrazine-induced DNA damage in the rat colon [120]. The antioxidant activity of propolis has also been connected to its protective effect against heavy metal toxicity [121], cardiovascular anomalies, and brain pathologies, as described in the above sections. An in vivo study conducted on 47 healthy men and women to evaluate the effects of propolis supplementation on antioxidative status and red blood cells revealed that as compared to women, men were more benefited by intake of propolis supplements and showed a significant reduction in lipid peroxidation [76]. Turkish propolis has been shown to inhibit H_2_O_2_-induced DNA damage in culture fibroblasts [122]. Antioxidant properties in propolis have been assigned to several of its components, including CAPE, quercetin, kaempferol, pinocembrin, flavones and flavonols [123,124]. Interestingly, an aqueous extract of propolis was reported to be more effective than the ethanol extracts due to the higher polyphenol content. The effect of pinocembrin on amyloid-beta (Aβ) accumulation, a key factor in Alzheimer’s disease pathology, was examined. APP/PS1 transgenic mice treated with pinocembrin for 3 months showed protection against the cognition decline without altering Aβ burden and oxidative stress. Instead, a significant level of neurovascular protection (maintenance of neuropil ultrastructure, preservation of microvascular function, reduction in glial activation and levels of inflammatory mediators) was observed [125]. CAPE was also shown to provide stress protection against oxidative stress induced by H_2_O_2_ and scopolamine in neuronal cell culture and animal models [126,127]. A study examined the effect of CAPE on methotrexate-induced testicular toxicity in rats. Whereas the level of lipid peroxidation was higher in the methotrexate group than in the control group, the CAPE-treated group showed decrease [128]. CAPE was found to stabilize hypoxia-inducible factor-1 (HIF-1α), a major transcriptional regulator of hypoxia (a condition of oxygen deprivation), by inhibiting factor inhibiting hypoxia (FIH), which inhibits HIF-1α by hydroxylation at ASN803 [129,130]. These studies have suggested the strong potential of propolis in modulation of oxidative stresses and protection against a variety of oxidative stress-related diseases.

### 3.6. Anticancer Activity

Cancer, a disease of uncontrolled cell proliferation, is a highly complex and heterogeneous disorder. In sharp contrast to normal cells, cancer cells escape the replicative senescence process and undergo infinite division and malignant transformation resulting in tumors (masses of cells) at any site of the body, and they often migrate from the primary site to secondary distant sites in the process of metastasis. Recently, it has been shown that aging and cancer are highly correlated, and that the incidence of cancer increases with age. Often, the innate tumor suppressor mechanisms that regulate cellular senescence are either lost or functionally inactivated in cancer cells. Hence, aging is considered as a pro-tumorigenic state that contributes to cancer development by physiological alterations in response to stress, DNA damage and inflammation [64]. Although significant and rapid developments have been witnessed in cancer diagnostics and treatment in the past decade, the highly invasive cancers still have high mortality and treatment failure and hence warrant new treatment modalities. Furthermore, the current chemotherapeutic drugs are highly expensive (costing approximately USD 100,000 per person); cause toxicity and adverse side-effects, such as hormonal imbalance, hair loss, low immune response, fatigue and nausea; and are often limited by drug resistance [131,132]. In this context, natural compounds with anticancer activity have gained a focus of research and drug development. Indeed, over 60% of the current anticancer drugs were derived in one way or other from natural sources [133]. Taxol, vinblastine and camptothecin have been derived from plants and comprise a large portion of current-day pharmaceutical agents, most notably in the area of chemotherapy. In addition to compounds from terrestrial plants, compounds from marine sources (e.g., ecteinascidin 743, halichondrin B and dolastatins), microbes (e.g., bleomycin, doxorubicin and staurosporin) and slime molds (e.g., epothilone B) have been shown to possess remarkable cancer chemotherapeutic potential [134]. Natural products have been shown to combat cancer by a variety of mechanisms. In several cases, they do not kill cancer cells directly, but boost the immune system to eliminate them [135]. Based on these aspects, mechanisms of anticancer activities in natural compounds have been gaining popularity in research. Many plant-derived polyphenols such as resveratrol, green tea polyphenols, piperine, flavopiridol, curcumin, genistein and caffeic acid phenethyl ester have been investigated for their cancer-preventive and chemotherapeutic properties [136,137]. Several studies have summarized the anticancer potential of natural bee products or their active components as an alternative treatment of human tumors [138]. Using a variety of human cancer-derived cell lines, several studies have reported anticancer activity in varieties of propolis from different geographical regions that vary in their bioactive constitution [139,140,141,142,143,144,145]. Furthermore, multimodal mechanisms of action for different propolis types and their components have been reported. These include immunomodulation and antitumor resistance [70,146,147], activation of apoptotic signaling [80,83,143,148,149,150], inhibition of cancer cell migration and invasion [143,151,152,153] and drug resistance [154,155].

The major components of propolis such as CAPE (New Zealand) and ARC (Brazilian) have been shown to cause cell-cycle arrest, inhibition of matrix metalloproteinases, cell migration, metastasis and angiogenesis. Galangin, cardanol, nemorosone, chrysin and other compounds of propolis are reported to block the rapid division of tumor cells. Several studies have reported the selective toxicity of CAPE and its derivative compounds on cancer/transformed cells [138,143,152,156,157]. CAPE was shown to inhibit 12-O-tetradecanoylphorbol-13-acetate (TPA)-induced tumors and therefore proposed as an anticancer drug for the treatment of gastric carcinoma, hepatoma and colorectal carcinoma [138,158]. The studies on a wide range of human cancer cell lines have reported that CAPE causes cytotoxicity to most with IC50 of 10–20 μM; some cell lines showed high sensitivity (IC50 1–5 μM), whereas some others showed low sensitivity (IC50 70–100 μM) [143,150,151,152].

**Figure 1:**
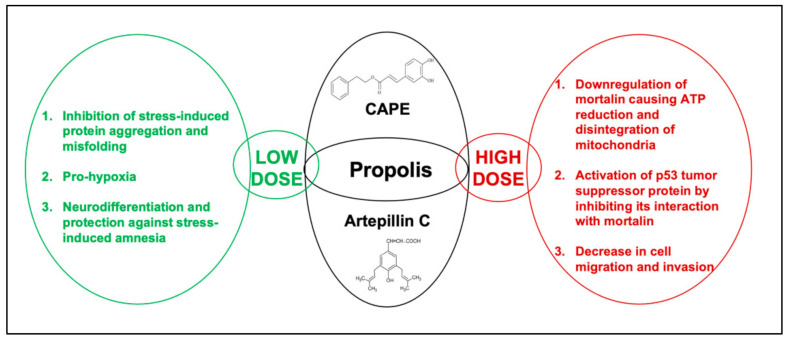
Schematic diagram showing the effect of low and high doses of propolis extract (CAPE and artepillin C, in particular) on cell proliferation, stress-induced protein aggregation and protein misfolding.

Mortalin/GRP75, a mitochondrial chaperone, is enriched in a large variety of cancer cells. It has been shown to contribute to carcinogenesis in multiple ways, including inactivation of the function of tumor suppressor p53 protein, deregulation of apoptosis and promotion of cell migration and epithelial-to-mesenchymal transition (EMT) progression [150,159,160,161,162,163]. In several studies, the interaction of mortalin and p53 in cancer was found as a new therapeutic target for treatment. Several small molecules that downregulate mortalin or target mortalin–p53 interaction resulting in reactivation of tumor-suppressor p53 protein have been shown to possess anticancer potential [150,164,165,166,167,168]. On these lines, CAPE-treated cells showed growth inhibition/apoptosis mediated by downregulation of mortalin at transcriptional and translational levels and activation of p53 function, which was supported by both computational and experimental approaches [143,150]. Since CAPE was found to be unstable and degraded by secreted esterases, a CAPE–γ-cyclodextrin (γCD; produced from starch by the process of enzymatic conversion and well known for its use in pharmaceutical formulation of oral products to increase the bioavailability of poorly water-soluble or unstable drugs) complex was prepared [143,152]. In vitro and in vivo experiments demonstrated an enhanced anticancer activity of the CAPE–γ-cyclodextrin complex [143,152]. Various stress conditions such as high temperature and metal stress are known to cause protein misfolding and aggregation by shifting the conformation equilibrium more towards the aggregation state due to protein–protein interaction through their hydrophobic regions [169,170]. We investigated the antistress potential of nontoxic doses of CAPE by recruiting arsenite and high-temperature-induced protein aggregation in cells exogenously transfected with either green fluorescence protein (GFP) or luciferase proteins, respectively [130]. The system allowed direct observations of protein aggregation. Interestingly, we found that whereas control cells possessed expected diffused cytoplasmic fluorescence of GFP protein, arsenite-treated cells showed its strong aggregation. Protein deaggregation was seen in the cells recovered in CAPE-supplemented medium. In a similar assay, heat-induced aggregation of luciferase was used as a model. Whereas heat-induced aggregation of luciferase led to a decrease in quantitative reporter assays, cells treated with CAPE showed recovery (Figure 1). Furthermore, low nontoxic doses of CAPE that showed antistress and protein deaggregation activities also showed prohypoxia effect [130], suggesting its use in the treatment of metal and hypoxia stress-driven pathologies.

Brazilian propolis has been shown to activate the immune system and possess direct antitumor activity [171]. Artepillin C (ARC) was shown to exhibit a cytotoxic effect and inhibit the growth of human and murine malignant tumor cells in vitro and in vivo. It was also found to cause significant damage to solid tumors and leukemic cells [83,101]. Apoptosis, abortive mitosis and massive necrosis were identified by histological observation after intratumor injection of 500 mg/kg BW of ARC three times a week. Cytotoxic effects of ARC were most noticeable in carcinoma and malignant melanoma in human tumor cells xenografts grafted and transplanted into nude mice [83,139,152]. Similar to CAPE, ARC has been shown to activate p53 tumor-suppressor protein by abrogating its complex with mortalin (p53-inactivating protein) possessing the anticancer activity [130] (Figure 1). However, as compared to CAPE, ARC showed low efficacy (IC50 275 µM), and hence a unique superficial extract of green propolis (GPSE) containing a multitude of bioactive components was generated. As expected, GPSE exhibited higher cytotoxicity in in vitro cytotoxicity assays. Furthermore, its conjugate with γCD showed better activity both in in vitro and in vivo assays. The effects of low nontoxic doses of ARC and GPSE have not been reported as yet and warrant studies with suitable cell culture and animal models. Table 1 summarizes various bioactive components and bioactivities in different kinds of propolis discussed in the above sections.

## 4. Conclusions

Propolis has been used in folk medicines for centuries. Due to various therapeutic and health-beneficial activities, it is among the top few natural products that have been used, maintained, utilized and popularized over a long period of time. Flavonoids, polyphenolic compounds, flavones, flavanones, phenolic acids and their esters are the pharmacologically active molecules of propolis. These active molecules have shown broad arrays of biological effects, such as antibacterial, antioxidant, anti-inflammatory, antifungal, anticancer and antihypertensive activities. Propolis has been extensively investigated for its various biological and health-beneficial properties, and it is attracting further interest among researchers aiming to further understand the activities and mechanism(s) of action of propolis alone and in complex with other compounds. There has been an increase in consumer interest in propolis-containing products that has led to the development of newer products in the health supplement and cosmetic industries. The traditional knowledge of the utilization of propolis by the diverse indigenous populace offers substantial support to its health and therapeutic merits. However, precise and targeted drug development is yet to take place. The foremost requirement for drug development involves preclinical investigations using defined resources of propolis from diverse phytogeographical locations and its constituents in defined and established extraction protocols. With these biotechnological and pharmacological advances, propolis, its defined extracts and its active components have the potential to serve as useful resources for natural, economical and easily available therapeutic drugs for stress, age-related pathologies and cancer.

## Figures and Tables

**Table 1:** Types of propolis and their active components, bioactivities and mechanisms.

Propolis (Types)	Active Components	Bioactivities	Mechanisms	Refs.
New Zealand	Caffeic acid Caffeic acid phenethyl ester Quercetin Naringenin Flavanones Flavones Phenolic acids	Anti-inflammatory	Immunosuppression and inhibitor of early and late events in T-cell receptor-mediated T-cell activation NF-kB blocking Inhibition of NADPH oxidase and tyrosine-protein kinase Suppression of lipoxygenase pathway of arachidonic acid metabolism	[65,66,67,68,69,70,71,72,73,74]
		Antioxidant	Free radical scavenging activity HIF-1α stabilization and stress protection Reduction in hyperthermia-induced survival inhibition, necrosis Production and maintenance of superoxide dismutase activity	[87,120,121,122,123,124,125,126,127]
		Antitumor	Inhibition of TPA, mortalin–p53 interaction	[138,139,140,141,142,143,144,145,156,157]
		Antimetastatic	Inhibition of vascular endothelial growth factor (VEGF) production	[151,152,153]
		Hepatoprotective	Upregulation of SOD, GPx enzymes	[88,89,90,91,92]
		Antibacterial	Inhibition of bacterial motility	[33,34,35,36,37,38,39,40]
		Antiviral	Inhibition of viral replication	[52,55,60,61]
Brazilian	3,5-diprenyl-4-hydroxycinnamic acid, 3-prenyl-4-dihydrocinnamoloxycinnamic acid 2,2-dimethyl-6-carboxyethenyl-2H-1-benzopyran Triterpenoids Moronic acid Anwuweizonic acid Betulonic acid Artepillin C PM-3 (3-(2-dimethyl-8-(3-methyl-2-butenyl)benzopyran)-6-propenoic acid) Baccharin Drupanin Lignans 3,4-dicaffeoylquinic acid Prenylated p-coumaric acid Isoflavones Terpenes	Anti-inflammatory	Inhibition of NF-_k_B by proinflammatory cytokines suppression	[65,66,67,68,69,70,71,72,73,74]
		Antioxidant	Free radical scavenging activity	[120,125]
		Antitumor	Activation of immune system Activation of p53 Inhibition of migration	[139,143]
		Antiviral	Anti-HIV activity in H9 lymphocytes	[49,53]
		Hepatoprotective	Upregulation of SOD, GPx enzymes	[66,88]
		Antimicrobial	Antimicrobial activity against *Bacillus cereus*, *Enterobacter erogenous* and *Arthroderma benhamiae* Bacteriostatic, bactericidal activity	[33,34,35,36,37,38,39,40]
Taiwanese	Prenylated flavanones Propolin A, B and C	Antioxidant	Free radical scavenging activity	[141]
		Antitumor	Induction of apoptosis in human melanoma cells Inhibition of xanthine oxidase activity	[141]
Chinese	Flavones Flavanones Flavans	Antiproliferative effect	Antiproliferative activity towards colon 26-L5 carcinoma cells	[141]
			Cytotoxicity in cervical cancer	[78,141]
Canadian	Chalcones	Radical scavenging activity	Radical scavenging activity against DPPH	[5]
Greek	Flavanones Terpenes	Antimicrobial	Antimicrobial activity against Gram-positive and Gram-negative bacteria, pathogenic fungi	[3]
Cuban	Isoflavonoids Prenylated benzophenones Terpenes	Antioxidant	Free radical scavenging activity	[87]

## Data Availability

Not applicable.
